# Occurrence and prevalence of *Cronobacter* spp. in plant and animal derived food sources: a systematic review and meta-analysis

**DOI:** 10.1186/s40064-015-1324-9

**Published:** 2015-09-24

**Authors:** Norrakiah Abdullah Sani, Olumide A. Odeyemi

**Affiliations:** Food Safety and Quality Research Group, School of Chemical Sciences and Food Technology, Faculty of Science and Technology, National University of Malaysia, Bangi, Malaysia; Ecology and Biodiversity, Institute for Marine and Antarctic Studies, University of Tasmania, Hobart, Australia

**Keywords:** Prevalence, Reservoir, Contamination routes, Microbial food safety

## Abstract

*Cronobacter* species are motile, non-spore forming, Gram negative emerging opportunistic pathogens mostly associated with bacteremia, meningitis, septicemia, brain abscesses and necrotizing enterocolitis in infected neonates, infants and immunocompromised adults. Members of the genus *Cronobacter* are previously associated with powdered infant formula although the main reservoir and routes of contamination are yet to be ascertained. This study therefore aim to summarize occurrence and prevalence of *Cronobacter* spp. from different food related sources. A retrospective systematic review and meta-analysis of peer reviewed primary studies reported between 2008 and 2014 for the occurrence and prevalence of *Cronobacter* spp. in animal and plant related sources was conducted using “*Cronobacter* isolation”, “*Cronobacter* detection” and “*Cronobacter* enumeration” as search terms in the following databases: Web of Science (Science Direct) and ProQuest. Data extracted from the primary studies were then analyzed with meta-analysis techniques for effect rate and fixed effects was used to explore heterogeneity between the sources. Publication bias was evaluated using funnel plot. A total of 916 articles were retrieved from the data bases of which 28 articles met inclusion criteria. *Cronobacter* spp. could only be isolated from 103 (5.7 %) samples of animal related food while 123 (19 %) samples of plant related food samples harbors the bacteria. The result of this study shows that occurrence of *Cronobacter* was more prevalent in plant related sources with overall prevalence rate of 20.1 % (95 % CI 0.168–0.238) than animal originated sources with overall prevalence rate of 8 % (95 % CI 0.066–0.096). High heterogeneity (*I*^2^ = 84) was observed mostly in plant related sources such as herbs, spices and vegetables compared to animal related sources (*I*^2^ = 82). It could be observed from this study that plant related sources serve as reservoir and contamination routes of *Cronobacter* spp.

## Background

*Cronobacter* spp. are an emerging Gram-negative opportunistic pathogens mostly associated with neonatal infections such as infant meningitis, septicemia, bacteremia and necrotizing enterocolitis (NEC). They are formerly thought to be single species, called *Enterobacter sakazakii* (Ghassem et al. 2011; Hunter and Bean 2013). As a genus, *Cronobacter* belongs to the family Enterobacteriaceae. Members of the genus are motile, flagellated, non-spore formers, facultative anaerobes. The current seven members of the genus are: *C. sakazakii, C. turicensis, C. malonaticus, C. muytjensii,C. universalis, C. dublinensis andC. condimenti* (Joseph et al. 2012). Initially, powdered infant formula was thought to be the major source of these pathogens since the product is not sterile, however, studies have shown that *Cronobacter* spp. are ubiquitous opportunistic pathogens (Pagotto and Farber 2009; Asato et al. 2013; Craven et al. 2010; Lehner et al. 2010; Mozrová et al. 2014; Mullane et al. 2008). They have been isolated from different sources ranging from animal related food sources like powdered infant formula, follow up formula and plant related food sources like vegetables, herbs and spices (Joseph et al. 2012; Althaus et al. 2012; El-Sharoud et al. 2009; Hochel et al. 2012; Molloy et al. 2009; O’Brien et al. 2009; Schmid et al. 2009). Due to the severity of infections of these pathogens and presence in different samples, various sources has been hypothesized as reservoir for *Cronobacter* in the past years. In a study conducted by Iversen and Forsythe (2003), the following plant related sources: wheat, rice, herbs and spices were concluded to be possible reservoir for these pathogens from which the bacteria are brought in contact to other food sources via cross contamination. Textile filters for exhaust air of milk powder-producing plant was also hypothesized by Jacobs et al. (2011) as reservoir. Additionally, Lou et al. (2014) in a recent study also concluded wheat flour as natural reservoir and/or transmission route for *Cronobacter* spp. It could therefore not be ascertained if plant or other source serve as reservoir and contamination routes of these pathogens.

Meta-analysis is a quantitative and summarizing statistical techniques aimed at extracting and combining scientific results from multiple primary studies investigating the same research question (Gonzales-Barron et al. 2013). According to Sutton et al. (2001), the primary aim of meta-analysis is production of precise estimate of the effect size of a particular treatment, with increased statistical power, rather than using a single study. Meta-analysis can also explain possible differences in the study outcomes of primary studies by coding study characteristics like research design features, data collection procedures, type of samples or even year (Hox and Leeuw 2003). Meta-analysis involves several steps. Gonzales-Barron and Butler (2011a) suggested systematic review of literatures, data extraction of both qualitative and quantitative information from relevant primary studies, selection of effect size as described from each study, estimation of overall effect size of all the primary studies, assessment of heterogeneity of studies and presentation of meta-analysis using numerical (odd ratios, fixed effects size, p values, publication bias, meta regression, and random effect) and or graphical methods forest plot, funnel plot and others). In any scientific study, researchers can either perform experiment to generate data or utilize available data from previous study (primary study) without experimental work (den Besten and Zwietering 2012). Data from primary studies available in databases are usually used in meta-analysis. Until recently, only few meta-analytical studies have been conducted in food safety research as most meta-analysis are conducted in medical and social sciences. However, meta-analysis may be conducted in food safety research to proffer answers and solutions to mirage of research questions such as outbreak of food borne diseases, prevalence of microbial pathogens in foods, effect of pre- and post-harvest interventions, risk ranking of pathogens and consumer knowledge, attitude and practices (Xavier et al. 2014).

To the best of our knowledge, no meta-analysis has been conducted on estimation of overall occurrence, prevalence and detection of *Cronobacter* spp. in animal and plant related sources has been carried out in order to gain insight to source(s) of reservoir for these bacterial pathogens. This study therefore aim to systematically review and summarize primary studies describing occurrence and prevalence of *Cronobacter* spp. isolated from animal and plant food related sources with a view to identifying the natural reservoir and transmission routes of *Cronobacter* to other food sources.

## Methods

This study was carried out following suggested steps of Gonzales-Barron and Butler (2011a). The steps consist of systematic review of literatures, data extraction of both qualitative and quantitative information from relevant primary studies, selection of effect size as described from each study, estimation of overall effect size of all the primary studies, assessment of heterogeneity of studies and meta-analysis representation of obtained result using numerical (odd ratios, fixed effects size, p values, publication bias, meta regression, and random effect) and or graphical methods forest plot, funnel plot and others).

### Literature search, selection and relevance screening

A comprehensive literature search and systematic review of available primary studies aimed at producing summary of relevant, quality and initial findings from such studies. A problem statement describing the occurrence and prevalence of *Cronobacter* spp. in different samples to establish possible reservoir and routes of transmission of the pathogens was formulated to guide this study while the population considered in this study were various primary studies describing isolation of *Cronobacte*r in plant and animal food related sources. Presence and absence of the bacteria under consideration were considered as possible outcome of each primary study. Following the establishment of problem statement, population and outcome, comprehensive search of electronic databases (ISI Web of science and ProQuest) was carried out using the following search algorithms: “*Cronobacter* isolation”, “*Cronobacter* detection” and “*Cronobacter* enumeration”.

Titles and abstracts of retrieved primary studies were examined for eligibility and relevance to the study. Thereafter, full text articles of eligible primary studies were obtained from the databases. Articles that are not freely available were obtained via pay per view service of the National University of Malaysia’s library. Relevance of each article was screened using both inclusion and exclusion criteria. The inclusion criteria are isolation of *Cronobacter* from animal and plant originated food sources, description of isolation procedure, articles in English, full text and peer reviewed articles, number of samples and number of samples that are positive for presence of *Cronobacter* and total population are stated in the study while exclusion criteria are: review articles, detection of *Cronobacter* in artificially contaminated samples, non-peer reviewed articles such as thesis, opinion articles, non-food related sources of *Cronobacter* such as clinical samples and conference abstract due to lack of access to full articles. Articles were further checked for duplication using Endnote 5 software.

### Data extraction and assessment of quality

Modified method of Xavier et al. (2014) was used to extract data from the primary selected studies. The number of samples examined for presence of the bacteria and number of positive outcome required to find out the event rate of the samples were obtained from data extracted from the following 28 primary studies (Li et al. 2014; Jaradat et al. 2009; Baumgartner et al. 2009; Kandhai et al. 2010; Lee et al. 2012; Lou et al. 2014; Cetinkaya et al. 2013; Hochel et al. 2012; Terragno et al. 2009; Pan et al. 2014; El-Gamal et al. 2013; Chap et al. 2009; Wang et al. 2012). Characteristics of each primary study is summarized in Table 1.

**Table 1:** Characteristics of primary research studies

Study	Sample	No of sample	No of samples positive	References
Plant derived food samples				
Study 1	Herbs	22	1	Li et al. (2014)
Study 2	Herbs	67	26	Jaradat et al. (2009)
Study 3	Herbs	23	14	Baumgartner et al. (2009)
Study 4	Spices	28	1	Kandhai et al. (2010)
Study 5	Spices	26	7	Baumgartner et al. (2009)
Study 6	Cereal and cereal products	85	12	Li et al. (2014)
Study 7	Cereal and cereal products	123	6	Kandhai et al. (2010)
Study 8	Cereal and cereal products	50	8	Lee et al. (2012)
Study 9	Fresh vegetable produce	47	2	Kandhai et al. (2010)
Study 10	Ready to eat food	128	19	Lee et al. (2012)
Study 11	Fruits	41	3	Lee et al. (2012)
Study 12	Wheat	13	13	Lou et al. (2014)
Study 13	Dry noodles	5	5	Lou et al. (2014)
Study 14	Rice flour	7	2	Lou et al. (2014)
Study 15	Rice flour	12	4	Cetinkaya et al. (2013)
Study 16	Confessionary	42	3	Baumgartner et al. (2009)
Animal derived food samples				
Study 17	Powdered infant formula	32	1	Hochel et al. (2012)
Study 18	Powdered infant formula	3	3	Terragno et al. (2009)
Study 19	Powdered infant formula	76	1	Jaradat et al. (2009)
Study 20	Powdered infant formula	399	49	Pan et al. (2014)
Study 21	Powdered infant formula	140	20	El-Gamal et al. (2013)
Study 22	Follow up formula	5	1	Kandhai et al. (2010)
Study 23	Follow up formula	91	3	Chap et al. (2009)
Study 24	Powdered instant products	182	1	Kandhai et al. (2010)
Study 25	Milk powder	175	7	Kandhai et al. (2010)
Study 26	Pork	92	2	Wang et al. (2012)
Study 27	Minced meat	222	7	Kandhai et al. (2010)
Study 28	Powdered infant formula	395	8	Kandhai et al. (2010)

### Statistical analysis of extracted data

Statistical analyses was carried out using Comprehensive Meta-Analysis (CMA) software while *P* values <0.05 were considered as statistically significant. Fixed effects model was used to analyze combined extracted data while variation of occurrence and prevalence of *Cronobacter* spp. between the primary studies was evaluated using heterogeneity (*I*^2^). Presence of bias in the publication was determined using funnel plots (odd of presence of *Cronobacter* spp. in the samples) of standard error. Forest plots were however used to estimate the event rate at 95 % confidence intervals.

## Results

### Literature search

To the best of our knowledge, this study is the first meta- analytical study to be carried out with regard to these group of bacteria. The study was limited to 2008–2014 because many of the studies on isolation and characterization of *Cronobacter* were carried within these years. Likewise, it was between these years that the taxonomy and grouping of the bacteria into genus was carried out. As it could be seen in Table 1, only few primary studies met the inclusion requirement of this meta-analysis. The primary studies considered in this meta-analysis described standard method for isolation and detection of *Cronobacter* from the samples.

**Fig. 1:**
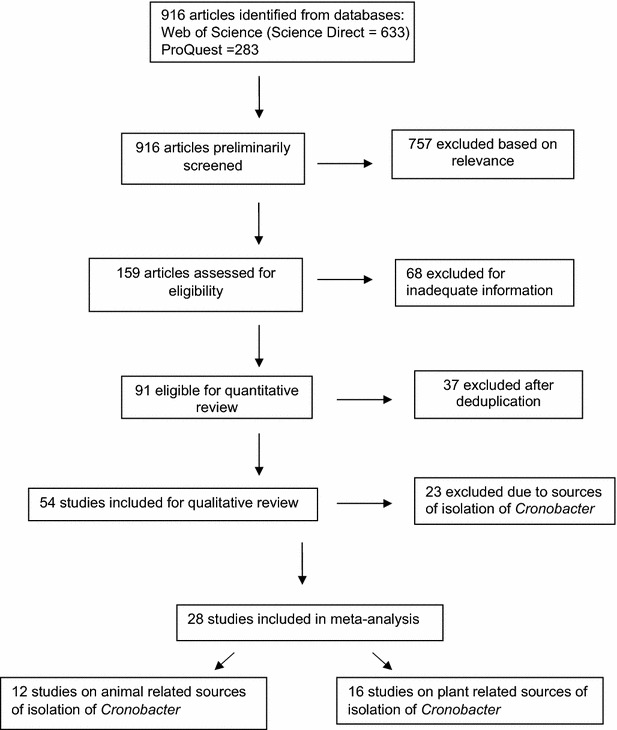
Flow chart outlining systematic review of primary studies

The primary studies were broadly grouped into plant originated/derived food samples and animal originated/derived food samples. In this study, systematic review of articles on primary studies was able to retrieve 916 articles (Web of Science -Science Direct = 633 and ProQuest = 283 respectively; 757 excluded based on relevance to study objectives while 159 articles were assessed for eligibility. Sixty-eight articles further excluded for inadequate information and 91 articles were eligible for qualitative review. Thereafter, 37 primary studies were excluded after deduplication. Twenty-eight (28) comprising of 12 studies on animal related sources of isolation of *Cronobacter* and 16 studies on plant related sources of isolation of *Cronobacter* were included in meta-analysis (Fig. 1). The six group of animal derived food samples in the 12 primary studies are powdered infant formula (1045 samples), follow formula (96 samples), powdered instant products (182 samples), milk powder (175 samples), pork (92 samples) and minced meat (222 samples) while the 10 group of plant derived food samples in the 16 primary studies are herbs (112 samples), spices (54 samples), cereals and cereal products (258 samples), fresh vegetables produce (47 samples), ready to eat foods (128), fruits (41 samples), wheat (13 samples), dry noodles (5 samples), rice flour (12 samples) and confessionary (42 samples) respectively hence, a total of 1812 of animal originated food samples and 644 plant related food samples were analyzed in all the primary studies. However, *Cronobacter* spp. could only be isolated from 103 (5.7 %) samples of animal related food while 123 (19 %) samples of plant related food samples harbours the bacteria.

### Prevalence of *Cronobacter* spp. in plant and animal originated food samples

**Fig. 2:**
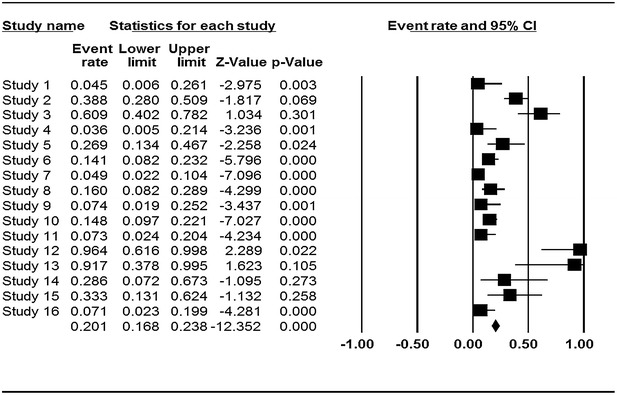
Forest plots of prevalence of *Cronobacter* spp. in plant originated food samples for fixed effects meta-analyses (*squares* represent effect estimates of individual studies with their 95 % confidence intervals of prevalence with size of squares proportional to the weight assigned to the study in the meta-analysis). The *diamond* represents the overall result and 95 % confidence interval of the fixed-effects meta-analysis

**Fig. 3:**
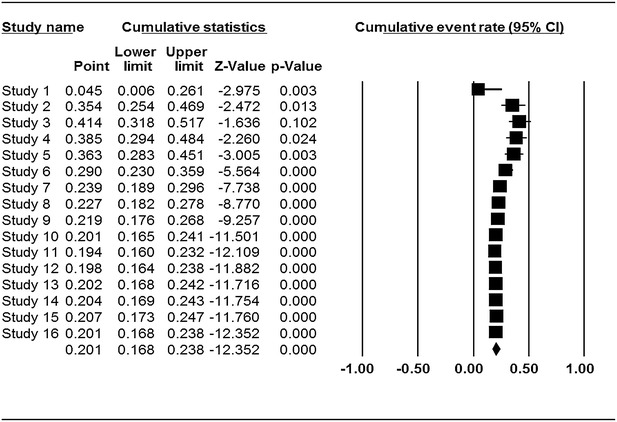
Cumulative meta-analysis of prevalence of *Cronobacter* spp. in plant originated food samples

**Fig. 4:**
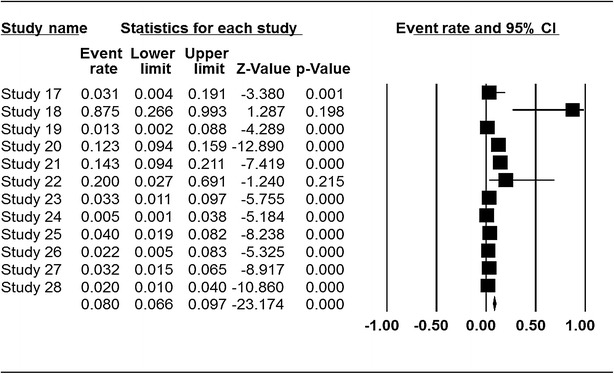
Forest plots of prevalence of *Cronobacter* spp. in animal originated food samples for fixed effects meta-analyses (*squares* represent effect estimates of individual studies with their 95 % confidence intervals of prevalence with size of squares proportional to the weight assigned to the study in the meta-analysis). The *diamond* represents the overall result and 95 % confidence interval of the fixed-effects meta-analysis

The characteristics of the primary studies summarizing the type of samples, occurrence and prevalent rates of *Cronobacter* used in this systematic review and meta-analysis is outlined in Table 1. The result of this study shows that occurrence of *Cronobacter* was more prevalent in plant related sources with overall prevalence rate of 20.1 % (95 % CI 0.168–0.238) as seen in the forest plot (Fig. 2) depicting forest plot of prevalence of *Cronobacter* spp. in plant originated food samples for fixed effects meta-analyses. Squares represent effect estimates of individual studies with their 95 % confidence intervals of prevalence with size of squares proportional to the weight assigned to the study in the meta-analysis. The diamond represents the overall result and 95 % confidence interval of the fixed-effects meta-analysis. The cumulative forest plot (Fig. 3), is the meta-analysis of all the primary studies in relation to each other. It displays results of mean prevalence rate of *Cronobacter* spp. from plant originated food samples by cumulatively adding one primary study at a time to another. In our study, overall prevalence rate of animal originated sources was 8 % (95 % CI 0.066–0.096) indicated by the forest plot (Fig. 4) which depicts prevalence of *Cronobacter* spp. in animal originated food samples for fixed effects meta-analyses. Of all the 12 primary studies included in this study on animal originated food samples, only samples (3) analyzed in study 18 showed high prevalence rate of 87.5 % (95 % CI 0.266–0.993) in comparison with the total samples (3) of powdered infant formula analyzed in the study.

### Publication bias among the primary studies

**Fig. 5:**
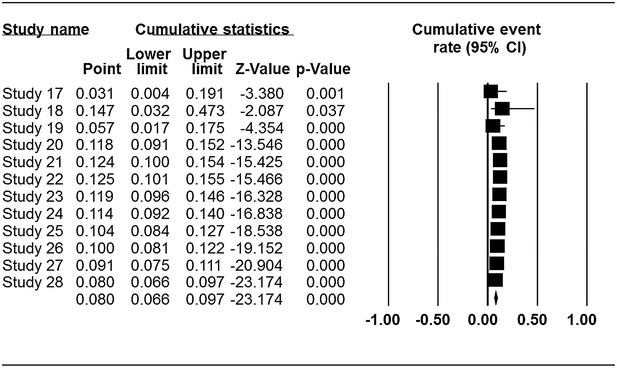
Cumulative meta-analysis of prevalence of *Cronobacter* spp. in animal originated food samples

Publication bias and quality of primary studies are limiting in meta-analysis (Noble 2006). Funnel plots are usually used to graphically assess publication bias in meta-analysis (Gonzales-Barron and Butler 2011b; Soon et al. 2012). This is obtained by plotting standard error against prevalence rate. In our study, publication bias could be observed among the primary studies due to asymmetric nature of the plots. Solid vertical line in the funnel plots represents the summary of prevalence rate derived from fixed-effect meta-analysis while the diagonal lines represent 95 % confidence interval. The cumulative forest plot (Fig. 5) of all the primary studies shows the mean prevalence rate of *Cronobacter* spp. from animal originated food samples by cumulatively adding one primary study at a time to another. Additionally, there is need for more studies on animal related sources of *Cronobacter* and the results of the research should be communicated in scientific publications. Some of the factors that can may possibly contribute to asymmetric funnel shape includes insufficient sample size, under reported primary research (Gonzales-Barron and Butler 2011b).

**Fig. 6:**
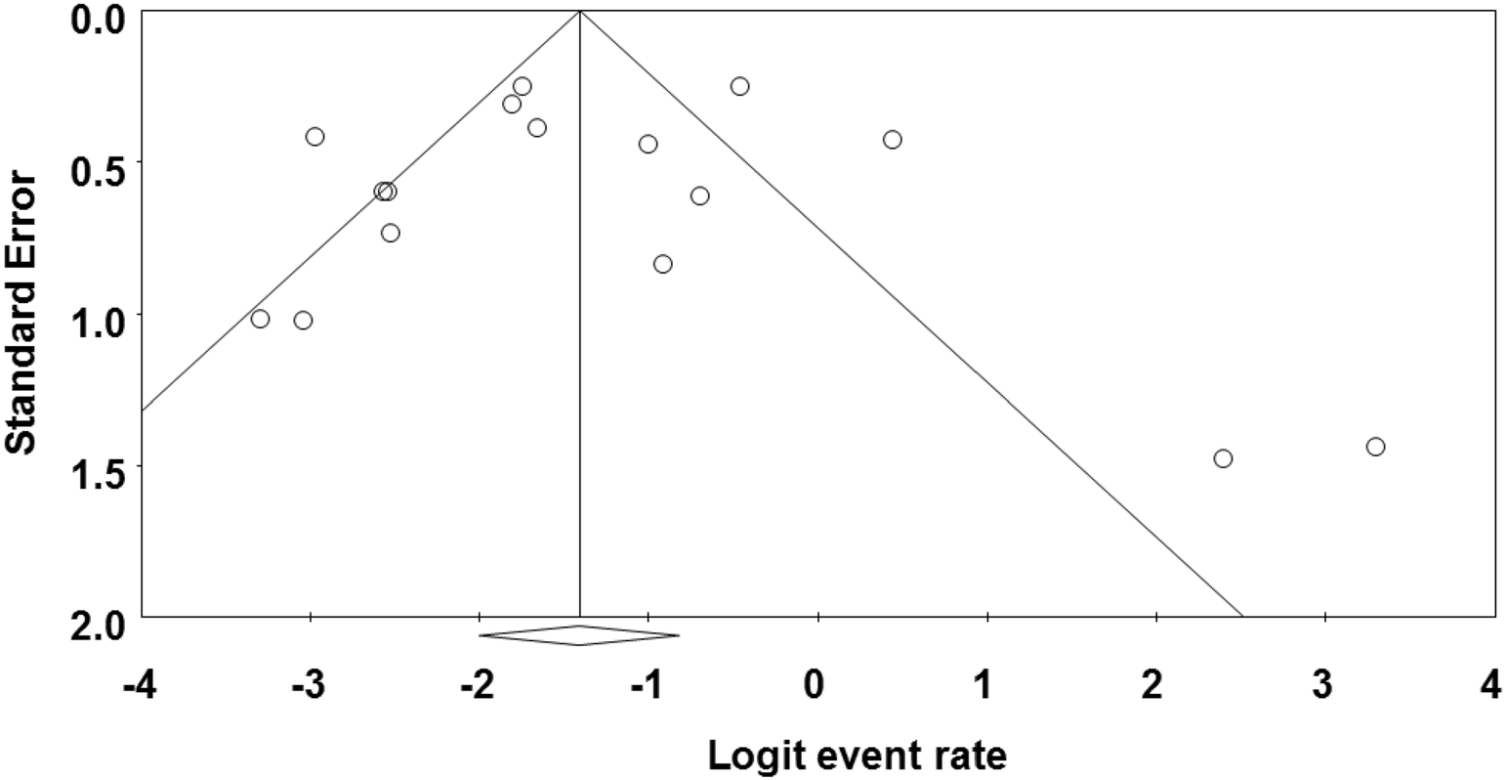
Funnel plot of prevalence of *Cronobacter* spp. in plant originated food samples. *Solid vertical line* represents the summary prevalence rate derived from fixed-effect meta-analysis while the *diagonal lines* represent 95 % confidence interval

**Fig. 7:**
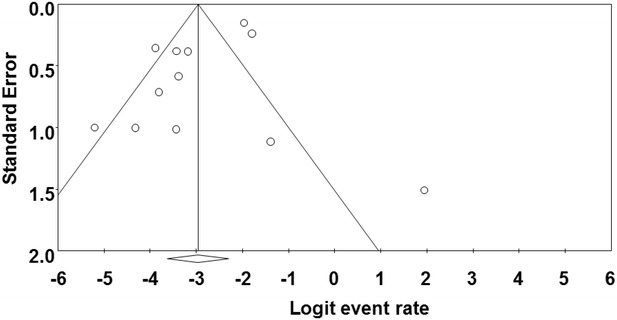
Funnel plot of prevalence of *Cronobacter* spp. in animal originated food samples. *Solid vertical line* represents the summary prevalence rate derived from fixed-effect meta-analysis while the *diagonal lines* represent 95 % confidence interval

In the funnel plots of prevalence of *Cronobacter* spp. in plant originated food samples (Fig. 6), most of the primary studies cluster at the top away from the effect size and toward the confidence interval at the left side of the plot between standard error 0.5 and 1. Likewise in Fig. 7, majority of the primary studies cluster at the top away from the effect size and toward the confidence interval at the left side of the plot between standard error 0.5 and 1. Publication bias among studies in Fig. 7 is higher than Fig. 6. More so, high heterogeneity (*I*^2^ = 84) was observed mostly in plant related sources such as herbs, spices and vegetables compared to animal related sources (*I*^2^ = 82) which correlates with the fact that more plant related food sources were included in this study than animal related samples.

## Discussion

Over the years, the number of studies on *Cronobacter* have increased. Despite this increase, the reservoir and routes of contamination of this bacteria is yet to be unanimously agreed upon by researchers. There is there need to conduct meta-analysis which quantitatively combine results of available primary studies with a view to identify the reservoir and possible contamination routes of *Cronobacter*. It is also used to explore any form of bias in the samples by cumulatively adding studies according to sample the number of samples that are positive for the presence of *Cronobacter* in relation to the sample size of each primary study. Although both summary effect forest plot and cumulative forest plot of primary studies on plant originated food look similar, in terms of the point estimate and confidence interval, however, it could be noted that cumulative forest plot showed persistence in the pattern of the obtained values. This was similar to the pattern of results obtained by Soon et al. (2012) in their study of meta-analysis of food handlers’ knowledge and attitudes towards food safety.

Variance in occurrence of *Cronobacter* between primary studies was observed in our study. The bacteria was more prevalent in samples analyzed in primary study 12 than others with prevalence rate of 96.4 % (95 % CI 0.616–0.998). This was as result of isolation of *Cronobacter* from all the 13 samples of wheat investigated in the study. Similarly, study 13 has high prevalence rate 91.7 % (95 % CI 0.378–0.995) as result of isolation of *Cronobacter* from all 5 samples of dry noodles examined in the primary study. It could be observed that both samples were analyzed in the same primary studies. There could possibly be some form of cross contamination during processing of the samples. However, this was not stated in the study. Contamination of food products constitute one of the major transmission routes for opportunistic foodborne pathogen (Lou et al. 2014). Studies have shown that *Cronobacter* could survive in food samples with low activity water. According to Osaili and Forsythe (2009), *Cronobacter* has various survival mechanisms including resistance to desiccation and osmotic stresses (Osaili and Forsythe 2009). These survival mechanisms will enable the persistence of the bacteria in the environment and dry food samples. The least plant derived food samples contaminated with *Cronobacter* is the study involving spices. Spices are also dry plant derived ingredients. However, considering the prevalence rate to other studies, it showed prevalent rate of 3.6 % (95 % CI 0.005–0.214).

Powdered infant formula has been initially thought to be the main source of *Cronobacter* due to the fact the products are not sterile however our study has shown that other sources could serve as reservoir and transmission routes to this opportunistic pathogens. Study 24 involving analysis of powdered instant products has the least prevalence rate of 5 % (95 % CI 0.001–0.038). It will be noted likewise that the results of study 18 and study 22 are statistically insignificant with p values >0.005. Both summary effect forest plot and cumulative forest plot of primary studies on animal originated food look similar, in terms of the point estimate and confidence interval, only study 18 shows large confidence interval while the remaining studies exhibited similar pattern down the study. Our current meta-analysis implies that more studies involving isolation of *Cronobacter* spp., from spices and other plant related sources need to be carried out.

## Conclusion

This current study is the first systematic review and meta-analysis to be conducted on occurrence and prevalence of *Cronobacter* in various samples. This outcome of this study supported some individual study stipulating that plant related food sources such as wheat, herbs, spices and flour could be potential reservoir and routes of contamination of *Cronobacter.* Although more animal related samples were analyzed than samples from plant related sources by the primary research studies included in the meta-analysis, occurrence of *Cronobacter* was more prevalent in plant related samples. Meta-analysis is without limitations. In our study, one of the possible limitation encountered is that only studies reported in English language were used. There could be possibility that positive results involving isolation of *Cronobacter* from animal related sources are reported. This correlates with the publication bias observed in the study which involve publication of study with significant results. Additionally, primary research studies involving clinical samples were not included in this study. It could be observed from this study that plant related sources serve as reservoir and contamination routes of *Cronobacter* spp., however more meta - analytical study to investigate prevalence of *Cronobacter* spp., among various samples within animal and plant sources.
